# Do Residents and Healthcare Providers Differ in Preference for Family Doctor Contract Service? Evidence From a Discrete Choice Experiment

**DOI:** 10.3389/fpubh.2022.800042

**Published:** 2022-02-10

**Authors:** Jiao Zhang, Lingzhong Xu, Wenzhe Qin, Aijun Xu

**Affiliations:** ^1^School of Health Economics and Management, Nanjing University of Chinese Medicine, Nanjing, China; ^2^Jiangsu Research Center for Major Health Risk Management and TCM Control Policy, Nanjing University of Chinese Medicine, Nanjing, China; ^3^Centre for Health Management and Policy Research, Cheeloo College of Medicine, School of Public Health, Shandong University, Jinan, China

**Keywords:** family doctor contract service, discrete choice experiment, preference, resident, healthcare provider

## Abstract

**Objective:**

Few are known on how and to what extent residents and healthcare providers have different preferences for family doctor contract service (FDCS). This study aimed to elicit and compare the residents' and healthcare providers' preferences for FDCS through a discrete choice experiment (DCE).

**Methods:**

Residents and healthcare providers recruited for the DCE were asked to choose repeatedly between two hypothetical service plans, which differed in six attributes: cost, service package, service delivery, type of service, accessibility of medicine, and level of healthcare team. We use mixed logit regression models to determine preferences for potential attributes.

**Results:**

A total of 2,159 residents and 729 healthcare providers completed valid DCE questionnaires. The mixed logit model results suggested that cost, service package, service delivery, type of service, accessibility of medicine, and level of healthcare team all had a significant impact on residents' and healthcare providers' preference. The level of healthcare team was the most important characteristic of FDCS to both residents and healthcare providers, followed by types of service. They have different preferences on the cost and way of service delivery.

**Conclusions:**

This study provides new evidence on how and to what extent residents and healthcare providers have different preferences for FDCS by determining their perception of various service attributes. These findings suggested that the optimal design and improvement of FDCS plans should consider not only residents but also healthcare providers' preferences to maximize contract service uptake.

## Introduction

General practice is regarded by the World Health Organization as the most economical and appropriate healthcare service model. International experience has proved that the promotion of family doctors contract service (FDCS) is an important way to strengthen the primary healthcare system and protect and maintain people health. The Chinese government has piloted the FDCS project since 2016 (1) and proposed that FDCS is healthcare services provided by signing service contract with family doctors (FDs) in the community healthcare centers, and signing service contract with FDs to use FDCS is voluntary. After years of practice, China has initially established a FDCS system and formed some service models with local characteristics; for example, Shanghai “1 + 1 + 1” contract service model (2), Hangzhou “integrated medical treatment and nursing care system” contracted service model (3). Previous studies have shown that the implementation of FDCS has generally improved the effectiveness of self-management in health (4) and primary care quality (5). But at the same time, there are some problems that restrict the progress of FDCSs in China. More recently, some researchers have noted that both the actual signing rate and utilization rate of FDCS are far from the national target in China and need to be improved. A meta-analysis study from Li et al. (6), for example, showed that the signing rate of FDCS for Chinese residents was 46.2% (95%CI: 35.5–56.9%). Further, Deng et al. (7) found that the overall utilization rate of FDCS was 6.9%. In addition to improve the supporting measures and guarantee mechanism at national level, optimizing the design of FDCS plans is also considered as a key determinant to comprehensively promote the quality and efficiency of FDCSs (8). The important prerequisite for this is to clarify the preferences for FDCS from the perspective of residents (demander) and healthcare providers (supplier) (9). However, evidence is unclear cut on how and to what extent residents and healthcare providers have different preferences.

Discrete choice experiments (DCEs) have been used to measure patient and healthcare providers preferences in a range of settings internationally (10). A DCE can be conducted to measure preferences for attributes of treatment by eliciting choices between hypothetical treatment profiles with systematic differences in their attributes (11). Using DCEs in primary care is valuable for determining how to improve rational shared decision-making. Including patient preferences when designing and evaluating healthcare programs can prove beneficial and help broaden the perspective on new or existing technologies. Therefore, this approach has been widely used in healthcare and health economic studies to quantify preferences for treatment attributes (12).

Prior researches have already estimated preferences using DCEs since the implement of FDCS in China. However, most of these studies focused on the view of the demander (residents/patients). For instance, Fu et al. (13) conducted a DCE in Chinese rural population and suggested residents valued the FDs' competence most. Zhu et al. (14) found that the most valued attribute in general practitioner (GP) care for patients was the organizational factors related to whether the provider had sufficient medicine and equipment to provide capable primary care service. Only one study to data measured the supply preferences from the perspective of healthcare providers (15). Meanwhile, similar studies have been conducted in other countries (16–20); nevertheless, considering that patients and healthcare providers references may be subjected to cultural and policy differences, the applicability of research from overseas to China mainland may be limited. More importantly, given the asymmetry of information between consumer and provider, it is not always clear that observed healthcare consumption is based on consumers' preferences and choice alone (21). It is important to understand that the value residents and healthcare providers place on different attributes of FDCS and how these preferences differ. To date, however, no study has been conducted to compare the demand and supply preferences of residents and healthcare providers for FDCS.

To make up for the research gap, the aim of this study is, therefore, to elicit and quantify residents and healthcare providers preferences for various attributes of FDCS by conducting DCEs and to explore the commonalities and differences between the demander and the supplier. We also examine the relative importance (RI) that patients and healthcare providers place on different treatment attributes. Results of this study could provide scientific evidences for the optimal design and strategic improvement in FDCS plans.

## Materials and Methods

According to the clear guidance on how to conduct DCEs proposed in previous literature (11, 22, 23), we developed and conducted the DCE in 4 main steps: (1) establishing attributes and levels for the experiment, (2) generating the experimental design and questionnaire, (3) collecting data, and (4) analyzing data.

### Establishing Attributes and Levels for the DCE

Identifying the attributes and levels that adequately describe the good or service of interest is the key step in DCE study. In our study, the service of interest was FDCS. The selected attributes and levels should be realistic and credible to residents and healthcare providers. We used a stepwise qualitative approach to establish attributes and levels for the DCE. First, we conducted a rapid literature review of existing DCE studies in primary healthcare and FDCS to select a preliminary list of attributes and levels. Combined with requirements of relevant policy documents of the FDCS in China, 10 important attributes were considered: content of service, the level of medical team, types of service, cost of contract, distance to practice, shared-decision making, insurance reimbursement rate, accessibility of medicine, ways of service, and attitude of service. Subsequently, we conducted semistructured interviews with five experts from research and practice (two researchers on FDCS, two GPs in primary healthcare institutions, and one DCE experts) and used the insights gained from these to validate and refine our selection of attributes and levels. Finally, six attributes that impact residents' and healthcare providers' decision-making the most were selected: cost of contract, content of service, types of service, ways of service, accessibility of medicine, and the level of medical team. The next step was to refine the terminology that described the attributes and levels. We chose levels for the cost attribute based on the spread of current prices for FDCS in China. The attributes 2, 3, 4, 5, and 6 were designed at 2 or 3 levels each to include the most common specifications of FDCS and capturing a realistic range within China's primary healthcare system. The final experimental design included six attributes with 2 or 3 levels each (see Table 1).

**Table 1:** Attributes and levels used in the DCE.

**Attributes**	**Levels**	**Description**
1. Cost	CNY10 ($65); CNY50 ($325); CNY100 ($650)	Annual out-of-pocket expenses for contracted services incurred by an individual resident
2. Service package	Basic package; Individualized package	The basic package includes national basic public health services and health management services; The individualized package includes basic package and personalized paid services for different groups of people
3. Service delivery	Outpatient visit; Telephone follow-up; Home visit	The ways of service provided by the contract medical team
4. Type of service	Chinese Medicine (CM); Western Medicine (WM); Integrated Chinese and Western Medicine (ICWM)	The types of service provided by the contract medical team
5. Accessibility of medicine	Low; Medium; High	The accessibility of medicine provided by the contract medical team.
6. Level of healthcare team	Level-I; Level-II; Level-III	Level-I refers to a core team composed of general practitioners or village doctors, community nurses, and public health personnel; Level-II is a horizontal combined team composed of the level-I team and specialists (assistants) in primary health institutions; Level-III is a vertical combined team composed of level-II team and experts from secondary and above medical institutions.

### Generating the Experimental Design and Questionnaire

Based on the attributes and levels we set, a large number of choice tasks will be generated (five attributes at three levels and one attributes at two levels = 3^5^*2^1^). To reduce the choice tasks to a manageable number, we used a fractional factorial design with 16 choice sets with two alternatives. We generated the experimental design using Stata 14.0 software, which chose a design based on optimal D-efficiency that allowed for the optimization of design efficiency, level balance, and the number of choice tasks. The DCE tasks were then divided into two blocks of eight choice sets each. Additionally, we included one repeated choice task as consistency test to ensure that each respondent made realistic trade-offs and to check internal validity. To reduce cognitive burden, respondents were randomly assigned to one of the blocks. To avoid larger numbers of respondents who choose the opt-out option to prevent making challenging choices, we did not leave respondents an opt-out option. This is also consistent with the policy background of our study: with the implementation of the policy of full coverage of FDCS, residents and healthcare providers must make their choice when they are assumed to participate in the FDCS. Previous study suggested that pictures were useful to explain attributes in a low-or middle-income country context where literacy cannot be assumed (24). Thus, we added visual elements into the questionnaire to reduce potential boredom and help respondents engage. To check the respondents' understanding of the questionnaire, a pilot survey was undertaken among 30 voluntary community residents and 10 healthcare providers in Tai'an city. We made minor changes to the format and layout, and our questionnaire was thought to be appropriated in length and understood easily by respondents through the pilot study. Table 2 shows an example choice task for residents and healthcare providers. The DCE choice tasks faced by residents and healthcare providers were exactly the same, whereas they had to choose between two services 1 and 2 from their different perspectives (demander and supplier).

**Table 2:** Example of a DCE choice task.

	**Service 1**	**Service 2**
Cost	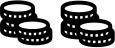 50 CNY	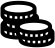 10 CNY
Service package	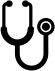 Basic	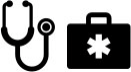 Individualized
Service delivery	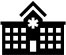 Outpatient visit	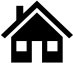 Home visit
Type of service	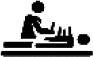 CM	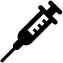 WM
Accessibility of medicine	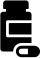 Low	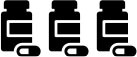 High
Level of healthcare team	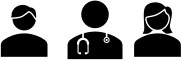 Level-III	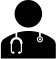 Level-I
Which one would you prefer?	□	□

In addition to the DCE part, the questionnaire for residents also included a series of questions concerning respondents' sociodemographic characteristics and current health situation, and the questionnaire for healthcare providers consisted of questions regarding sociodemographic characteristics, present work situation, and current health situation. All questionnaires included an explanation of the attributes and levels.

### Sampling and Data Collection

The survey was conducted in Tai'an city, Shandong Province, China. Multistage random sampling was used to choose the representative sample of residents and healthcare providers. First, three or four townships were randomly selected from each district (county) in Tai'an city; second, eight villages (communities) were randomly selected from each township, with a total of 160 villages (communities); third, residents were randomly selected from each village (community), and healthcare providers were enrolled from the selected primary health institutions. The inclusion criteria of residents were the key populations covered by FDCS: pregnant women, patients with chronic diseases, and the elderly aged 60 years and above. The inclusion criteria of healthcare providers were as follows: GPs, village doctors, nurses, public health workers, and other members of FDs team. We collected explicit and written consent from respondents after providing them with a detailed explanation of how their personal data would be used. To ensure the quality of the residents' responses, about 40 min of one-to-one, face-to-face interview for every participant was conducted using the questionnaire by the trained enumerator. Since most healthcare providers had high levels of educational attainment, a centralized self-filling questionnaire method was adopted, but two research assistants accompanied participants from commencement to the completion of the survey with assistance on any queries they may have.

The minimum required sample size for DCE, based on the method suggested by Orme (25), was 84 (500 × 3 ÷ 9 ÷ 2) respondents in this study. To increase precision of estimates, 2,226 residents and 816 healthcare providers were enrolled in DCE study, of which 67 residents and 87 healthcare providers were eliminated for failing consistency test, respectively. Finally, a total sample of 2,159 residents and 729 healthcare providers were included the statistical analysis.

### Data Analysis

We analyzed the choice observations from residents and healthcare providers separately using mixed logit model, which is a commonly used method for examining DCEs (26). In our model, expected overall utility U of respondent *i* from service plan *j* in the choice set *t* was given by:

$$\begin{array}{lll} U_{ijt} & = & \beta_{1i}Cost_{itj}+\beta_{2i}Package_{itj}+\beta_{3i}ServiceDelivery_{itj}\\ +\beta_{4i}Type_{itj}+\beta_{5i}Medicine_{itj}\\ +\beta_{6i}HealthcareTeam_{itj}+\varepsilon_{ijt} \end{array}$$

A significant coefficient (β) indicates that the attribute (level) is important for the participants' decision for FDCS. The utilities were converted into odds ratios (ORs) and a statistically significant OR (*p* < 0.05) indicated that the attribute level had an impact on the choice process of the participants. We calculated the RI of each attribute by computing the difference in the utility of the highest and lowest level of that attribute, divided by the sum of differences of all attributes. We additionally calculated the willingness to pay (WTP) of residents and willingness to supply (WTS) of healthcare providers by taking the ratio of the preference weight of the attribute to the preference weight of the cost of service. We carried out the entire data analysis using Stata 14.0 software.

## Results

### Characteristics of Participants

Characteristics of residents (*n* = 2,159) are reported in Table 3. The residents had a mean age of 63.06 years (SD = 10.76), the majority of them were women (58.04%), and 66.33% lived in rural area. A total of 79.99% have been diagnosed with one or more chronic diseases, and more than half of residents (56.97%) reported good health. Table 3 also shows the profile of healthcare providers (*n* = 729). The mean age of healthcare providers was 42.78 years, 47.33% of them were women, and 57.48% lived in rural area. More than half of them were worked in the village clinic (60.22%), <20% had the intermediate title or above, and 144 (19.75%) reported having chronic conditions.

**Table 3:** Characteristics of participants.

**Residents (*****n*** **= 2,159)**		**Healthcare providers (*****n*** **= 729)**	
**Variables**	***n* (%)**	**Variables**	***n* (%)**
Female	1,253 (58.04)	Female	345 (47.33)
Age, Mean±SD	63.06 ± 10.76	Age, Mean±SD	42.78 ± 8.55
Residence		Residence	
Rural	1,432 (66.33)	Rural	419 (57.48)
Urban	727 (33.67)	Urban	310 (42.52)
Marital status		Marital status	
Couple	1,800 (83.37)	Couple	670 (91.91)
Single	359 (16.63)	Single	59 (8.09)
Education		Education (year)	
Primary school and below	1,138 (52.71)	≤ 12	256 (35.12)
Junior school	666 (30.85)	13~15	310 (42.52)
Senior school and above	355 (16.44)	≥16	163 (22.36)
Annual household income (yuan)		Annual personal income (yuan)	
≤ 10,000	572 (26.49)	≤ 15,000	160 (21.95)
10,001~25,000	491 (22.74)	15,001~20,000	131 (17.97)
25,001~45,000	390 (18.06)	20,001~30,000	151 (20.71)
45,001~70,000	337 (15.61)	30,001~40,000	125 (17.15)
>70,000	369 (17.09)	>40,000	162 (22.22)
Chronic conditions		Workplace	
Yes	1,727 (79.99)	Community health center	127 (17.42)
No	432 (20.01)	Community health station	68 (9.33)
Self-rated health		Township health center	95 (13.03)
Good	1,230 (56.97)	Village clinic	439 (60.22)
Medium	31.08 (31.08)	Professional title	
Poor	11.95 (11.95)	None	283 (38.82)
		Junior	309 (42.39)
		Intermediate and above	137 (18.79)
		Chronic conditions	
		Yes	144 (19.75)
		No	585 (80.25)

### Discrete Choice Experiment Results

Table 4 presents the preferences of residents and healthcare providers. In general, all ORs were statistically significant, which suggests that all attributes played a role in their decision for demand and supply of FDCS. As expected, residents preferred to choose service with lower costs, and healthcare provider preferred to supply service with higher costs. Regarding the way of service delivery, healthcare providers preferred outpatient service instead of home visit, whereas residents were more likely to select home visit service. In addition, Integrated Chinese and Western Medicine (ICWM), a higher accessibility of medicine and higher level of healthcare team were preferred by residents and healthcare providers compared with the respective reference categories. However, neither residents nor healthcare providers preferred to pick individualized package. An analysis that includes participants who failed the consistency test provided highly similar results, which indicated that the preferences of residents and healthcare providers for FDCS was robust (see Supplementary File).

**Table 4:** Results of mixed logit model of residents and healthcare providers.

**Attribute levels**	**Residents**		**Healthcare providers**	
	**OR**	**95%CI**	**OR**	**95%CI**
Cost (per yuan ¥)	0.997^***^	(0.996, 0.998)	1.003^***^	(1.001, 1.004)
Service package (basic package ^a^)				
Individualized package	0.927^**^	(0.884, 0.972)	0.887^***^	(0.832, 0.946)
Service delivery (home visit ^a^)				
Outpatient visit	0.522^***^	(0.488, 0.559)	1.129^**^	(1.035, 1.231)
Telephone follow-up	0.540^***^	(0.511, 0.571)	1.019	(0.935, 1.111)
Type of service (CM ^a^)				
WM	1.067	(1.000, 1.138)	1.159^**^	(1.054, 1.275)
ICWM	1.711^***^	(1.621, 1.806)	1.386^***^	(1.275, 1.508)
Accessibility of medicine (low ^a^)				
High	1.465^***^	(1.383, 1.552)	1.135^**^	(1.043, 1.235)
Medium	1.577^***^	(1.477, 1.684)	1.073	(0.981, 1.173)
Level of healthcare team (level-I ^a^)				
Level-III	4.188^***^	(3.863, 4.540)	1.350^***^	(1.216, 1.499)
Level-II	2.522^***^	(2.374, 2.680)	1.408^***^	(1.285, 1.542)
ASC	1.039	(0.981, 1.101)	0.986	(0.909, 1.070)
No. of observations	34,544		11,664	
No. of respondents	2159		729	
Log likelihood	−9,764.399		−3,913.567	

*a, reference level; OR, odds ratio; CI, confidence interval; ASC, alternative special constant; CM, Chinese Medicine; WM, Western Medicine, ICWM, Integrated Chinese and Western Medicine;*

*** *p < 0.001;*

** *p < 0.01*.

**Figure 1:**
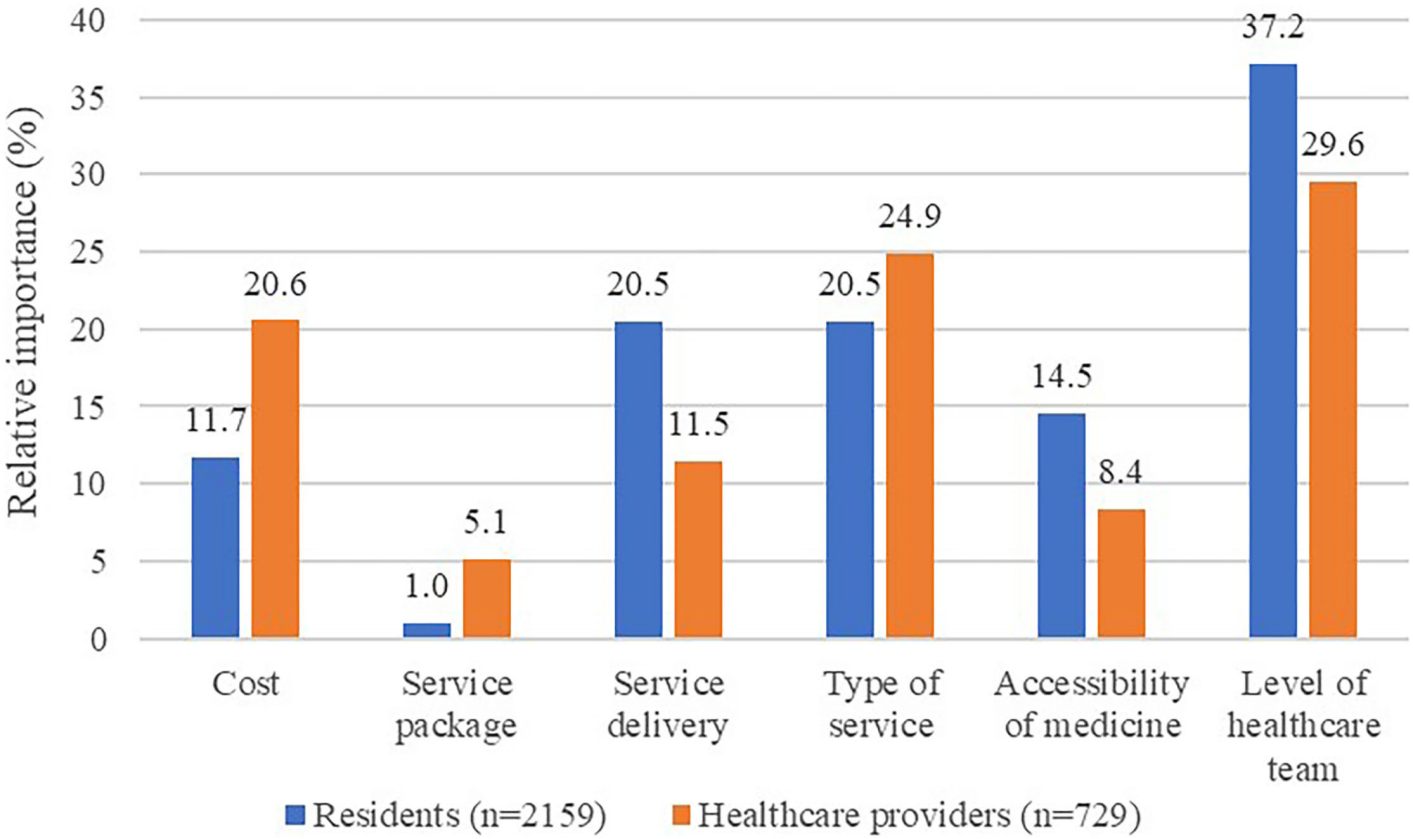
RI of the FDCS attributes for residents and healthcare providers. RI, relative importance; FDCS, family doctor contract service.

Figure 1 shows the RI of the attributes. Level of healthcare team was most important for both residents (37.2%) and healthcare providers (29.6%), followed by type of service. Moreover, healthcare providers placed more importance on cost (20.6%) and way of service delivery (11.5%). In contrast, residents valued way of service delivery (20.5%) and accessibility of medicine (14.5%). It was also noted that the service package was least important for both residents and healthcare providers, relative to all other attributes.

### Willingness to Pay and Willingness to Supply

Table 5 reports the WTP and WTS estimates based on the mixed logit model results. Residents were willing to pay CNY 490.44 (95%CI, 374.56–606.32) for their preferred highest level of healthcare team (yuan). This is higher than what they were willing to pay for the other attributes: Residents were willing to pay CNY 130.94 for high accessibility of medicine and CNY 183.95 to get ICWM service. The negative (–) results indicate theoretically to what extent the residents would be willing to be compensated for an attribute level. A subsidy of CNY 25.71 was required for residents to accept individualized package services. Meanwhile, the results showed that healthcare providers were willing to sacrifice certain service costs for the preferred attributes and levels: healthcare providers were willingness to sacrifice CNY 112.97 to supply the service by higher level of healthcare team, CNY 123.93 to provide ICWM service, and CNY 45.82 for outpatient service delivery. On the contrary, they would charge CNY 45.53 to provide individualized package service for contracted residents, instead of basic package service.

**Table 5:** WTP of residents and WTS of healthcare providers (yuan ¥).

	**WTP**	**(95%CI)**	**WTS**	**(95%CI)**
Service package				
Individualized package	−25.71^**^	(−42.68, −8.74)	45.53^**^	(73.62, 17.44)
Service delivery				
Outpatient visit	−222.26^***^	(−278.93, −165.59)	−45.82^*^	(−8.99, −82.64)
Telephone follow-up	−210.66^***^	(−262.98, −158.34)	−7.25	(−39.22, 24.72)
Type of service				
WM	22.12	(−0.22, 44.46)	−55.82^**^	(−90.11, −21.53)
ICWM	183.95^***^	(134.48, 233.41)	−123.93^***^	(−177.78, −70.08)
Accessibility of medicine				
High	130.94^***^	(93.67, 168.01)	−48.27^*^	(−87.49, −69.04)
Medium	156.16^***^	(115.81, 196.51)	−26.72	(−64.57, 11.14)
Level of healthcare team				
Level-III	490.44^***^	(374.56, 606.32)	−112.97^**^	(−180.76, −45.18)
Level-II	316.66^***^	(240.92, 392.41)	−130.02^***^	(−190.17, −69.86)

*WTP, willingness to pay; WTS, willingness to supply;*

*** *p < 0.001;*

** *p < 0.01;*

* *p < 0.05*.

## Discussion

To the best of our knowledge, this is the first study using a DCE to reveal the residents' and healthcare providers' preferences for FDCS and compare the difference in preferences between the demander and the supplier in China. Our study showed that cost of service, service package, way of service delivery, type of service, accessibility of medicine, and level of healthcare team all influenced residents' and healthcare providers' preferences for FDCS, which provides new insights on how residents and healthcare providers value attributes associated with FDCS from their different perspective.

Understanding the residents' preferences for FDCS could contribute to better service communication and quality to enhance uptake and adherence of FDCS. We found that residents were mostly driven by high level of healthcare team, and they had the highest WTP for the level-III healthcare team when choosing to sign up for the FDCS. This is not surprising, and the level of healthcare team is generally considered as representing appropriateness and quality of primary care, which could be linked with desired effect of care for patients. In line with this research, previous studies conducted in China revealed that respondents had a strong preference for the healthcare providers with high competence (13, 27, 28). However, recent evidence showed that the low competency of FDs was still one of the notable barriers to implement FDCS in China (8). FDs and team members are the flagbearers of FDCS, and their service capabilities, willingness, and attitudes all influence the quality of primary care. At the same time, the DCE results showed that healthcare providers also valued the higher level of healthcare team. Except the professional nature, which determines their instinct to provide better services to residents, altruistic behavior that has been proven in the previous studies may also explain these preference choices (29, 30). Moreover, results from previous studies have suggested FDs' role as perfect agent for their patients can be strengthened in the presence of an effective governance and operating environment (20, 31). Our results may contribute to the development of future policies taking into account the common preferences of residents and healthcare providers. Therefore, we recommend that more attention should be paid to not only the talent team construction of FDs and regular training, but also the general governance structure and regulation environment.

The type of ICWM service was another important driver of both residents' and healthcare providers' positive decision on FDCS plans. It has been proved that traditional Chinese medicine (TCM) can meet the needs for public health and primary medical care, improve health equity, and realize the great goal that everyone will get access to the basic medical and health services (32). Meanwhile, the ICWM theory for the prevention and treatment of chronic and infectious diseases is more widely accepted in China (33, 34). These highlight the need to promote ICWM services in the implementation and improvement of the FDCS.

Further, we also found different preferences in the way of service delivery between residents and healthcare providers. Similar to results from previous studies (16), our findings revealed that home visits significantly influenced the residents' preference for FDCS. The residents included in our study were key population, such as the elderly people, who generally have high healthcare needs and desire more home visit service. However, the characteristic of FDCS in China is that home visit services are provided by FDs only for those vulnerable group of old, multimorbid, and immobile persons who have specific needs (35). On the other hand, the reality is that the current number of primary healthcare providers is not inadequate (36), and the related laws and operating specifications for home visit services are not insufficient in China, which may explain why healthcare providers preferred the outpatient visit service generally. Policy interventions are needed to address these serious problems in the primary care system (e.g., shortage of FDs).

In general, choosing a service package that suits an individual's needs should be an important factor for signing up the FDCS program. However, our results showed that the service package, while relevant, was of the least importance to the residents and healthcare providers. These can partly be explained by the setting of attribute level in the DCE task. The way of the service package attribute was presented by only two levels, which may not have been as tangible as the difference between other attributes. This may have resulted in the small preference estimates we observed for the service package levels. In spite of this, we found both residents and healthcare providers preferred the basic package service instead of individualized package. This is probably owing to the current characteristics and status quo of Chinese FDCS implementation. The Chinese government has basically achieved full coverage of FDCS for key population; nevertheless, most residents instinctively and voluntarily signed up free basic package service, and the acceptance and uptake of paid individualized packages service among residents was still not high. This gives insight into the importance of improving the service package programs and strengthening the policy publicity to further promote the FDCS in China.

Our study findings and interpretations are subjected to certain limitations. As with other DCE studies, this study is subject to hypothetical bias, as respondents had to make choices between hypothetical service options. Second, only a limited number of attributes can be included in the DCEs. Nevertheless, we have attempted to present real-world decision-making environments by extensive literature review and qualitative interviews to ensure the relevance on FDCS. A third limitation of this study is that we did not provide respondents with an opt-out option, which may lead to parameter estimation bias, but this also requires more research to test. Finally, this DCE study was conducted in one city, which may limit the generalization of the findings to the whole country, and the follow-up study could expand the scope of sampling to verified the findings of this study.

## Conclusions

In this study, we identified residents' preferences for choosing FDCS plans, but also those of healthcare providers' preferences for supplying services. The high level of healthcare team, ICWM service, high accessibility of medicine, and basic package services were common preferences of residents and healthcare providers. Meanwhile, they have different preferences in the cost and way of service delivery. This information could help decision makers to set up appropriate FDCS programs to fit with residents' and healthcare providers' preferences. With the further advancement of China's FDCS programs, it is more important to consider the benefit-risk preferences that residents and healthcare providers have for different service attributes to optimize FDCS programs that can ultimately improve residents' health outcomes and healthcare providers' job satisfaction.

## Data Availability Statement

The raw data supporting the conclusions of this article will be made available by the authors, without undue reservation.

## Ethics Statement

Written informed consent was obtained from the individual(s) for the publication of any potentially identifiable images or data included in this article.

## Author Contributions

LX, JZ, and WQ: concept and design and acquisition of data. JZ and WQ: analysis and interpretation of data. JZ: drafting of the manuscript and statistical analysis. LX and AX: critical revision of the paper for important intellectual content, obtaining funding, and supervision. LX and JZ: provision of study materials or patients. LX and WQ: administrative, technical, or logistic support. All authors contributed to the article and approved the submitted version.

## Funding

This research was supported by the grants of National Natural Science Foundation of China (71974118) and National Social Science Foundation of China (No. 2018VJX065).

## Conflict of Interest

The authors declare that the research was conducted in the absence of any commercial or financial relationships that could be construed as a potential conflict of interest.

## Publisher's Note

All claims expressed in this article are solely those of the authors and do not necessarily represent those of their affiliated organizations, or those of the publisher, the editors and the reviewers. Any product that may be evaluated in this article, or claim that may be made by its manufacturer, is not guaranteed or endorsed by the publisher.
