# Supplementary material for: Do Residents and Healthcare Providers Differ in Preference for Family Doctor Contract Service? Evidence From a Discrete Choice Experiment
Source: Front Public Health. 2022 Feb 10;10:800042. doi: 10.3389/fpubh.2022.800042 (PMC8866243; doi:10.3389/fpubh.2022.800042)
Supplement: Supplementary file 1 [file Table_1.PDF]

Table S1 Results of sensitive analysis on preferences of residents

Attribute levels	Model I		Model II	
	Coefficient	*P*	Coefficient	*P*
Cost (per yuan ¥)	-0.003	＜0.001	-0.003	＜0.001
Service package (basic package [a])				
Individualized package	-0.075	0.001	-0.080	0.001
Service delivery (home visit [a])				
Outpatient visit	-0.631	＜0.001	-0.646	＜0.001
Telephone follow-up	-0.594	＜0.001	-0.607	＜0.001
Type of service (CM [a])				
WM	0.049	0.104	0.049	0.115
ICWM	0.527	＜0.001	0.544	＜0.001
Accessibility of medicine (low [a])				
High	0.377	＜0.001	0.386	＜0.001
Medium	0.426	＜0.001	0.438	＜0.001
Level of healthcare team (level-I [a])				
Level-III	1.378	＜0.001	1.413	＜0.001
Level-II	0.894	＜0.001	0.911	＜0.001
No. of respondents	2226		2159	
No. of observations	35616		34544	
Log likelihood	-10143.174		-9766.038	
AIC	20324.350		19570.070	
BIC	20485.480		19730.620	

a, reference level; Model I, include participants who failed the consistency test; Model II， exclude respondents who failed the consistency test.

Table S2 Results of sensitive analysis on preferences of healthcare providers

Attribute levels	Model I		Model II	
	Coefficient	*P*	Coefficient	*P*
Cost (per yuan ¥)	0.002	＜0.001	0.003	＜0.001
Service package (basic package [a])				
Individualized package	-0.117	＜0.001	-0.117	＜0.001
Service delivery (home visit [a])				
Outpatient visit	0.123	0.003	0.117	0.007
Telephone follow-up	0.022	0.572	0.014	0.725
Type of service (CM [a])				
WM	0.143	0.001	0.150	0.002
ICWM	0.316	＜0.001	0.324	＜0.001
Accessibility of medicine (low [a])				
High	0.128	0.001	0.124	0.003
Medium	0.086	0.035	0.075	0.087
Level of healthcare team (level-I [a])				
Level-III	0.287	＜0.001	0.303	＜0.001
Level-II	0.334	＜0.001	0.347	＜0.001
No. of respondents	816		729	
No. of observations	13056		11664	
Log likelihood	-4391.112		-3913.623	
AIC	8820.224		7865.245	
BIC	8962.287		8005.166	

a, reference level; Model I, include participants who failed the consistency test; Model II， exclude respondents who failed the consistency test.
